# *Bacillus anthracis* gamma phage lysis among soil bacteria: an update on test specificity

**DOI:** 10.1186/s13104-017-2919-8

**Published:** 2017-11-16

**Authors:** Cari B. Kolton, Nicole L. Podnecky, Sean V. Shadomy, Jay E. Gee, Alex R. Hoffmaster

**Affiliations:** 1 Centers for Disease Control and Prevention, U.S. Department of Health and Human Services, Atlanta, GA USA; 2 Present Address: Department of Pharmacy, Faculty of Health Sciences, UiT The Arctic University of Tromsø, Hansine Hansens veg 18, Tromsø, Norway; 3 Present Address: One Health Office, National Center for Emerging and Zoonotic Infectious Diseases, Centers for Disease Control and Prevention, Atlanta, GA 30333 USA; 4 Food and Agriculture Organization of the United Nations, Viale delle Terme di Caracalla, 00153 Rome, Italy

**Keywords:** *Bacillus anthracis*, Anthrax, Gamma phage

## Abstract

**Background:**

*Bacillus anthracis*, which causes anthrax in humans and animals, is enzootic in parts of the U.S. state of Texas where cases are typically reported in animals annually. The gamma phage lysis assay is a common diagnostic method for identification of *B. anthracis* and is based on the bacterium’s susceptibility to lysis. This test has been shown to be 97% specific for *B. anthracis*, as a small number of strains of other *Bacillus* spp. are known to be susceptible. In this study, we evaluated the performance of a combination of *B. anthracis* diagnostic assays on 700 aerobic, spore-forming isolates recovered from soil collected in Texas. These assays include phenotypic descriptions, gamma phage susceptibility, and real-time polymerase chain reaction specific for *B. anthracis*. Gamma phage-susceptible isolates were also tested using cell wall and capsule direct fluorescent-antibody assays specific for *B. anthracis*. Gamma phage-susceptible isolates that were ruled out as *B. anthracis* were identified by 16S rRNA gene sequencing.

**Findings:**

We identified 29 gamma phage-susceptible isolates. One was confirmed as *B. anthracis*, while the other 28 isolates were ruled out for *B. anthracis* by the other diagnostic tests. Using 16S rRNA gene sequencing results, we identified these isolates as members of the *B. cereus* group, *Bacillus* sp. (not within *B. cereus* group), *Lysinibacillus* spp., and *Solibacillus silvestris.* Based on these results, we report a specificity of 96% for gamma phage lysis as a diagnostic test for *B. anthracis,* and identified susceptible isolates outside the *Bacillus* genus.

**Conclusions:**

In this study we found gamma phage susceptibility to be consistent with previously reported results. However, we identified non-*B. anthracis* environmental isolates (including isolates from genera other than *Bacillus*) that are susceptible to gamma phage lysis. To date, susceptibility to gamma phage lysis has not been reported in genera other than *Bacillus*. Though these isolates are not of clinical origin, description of unexpected positives is important, especially as new diagnostic assays for *B. anthracis* are being developed based on gamma phage lysis or gamma phage proteins.

**Electronic supplementary material:**

The online version of this article (10.1186/s13104-017-2919-8) contains supplementary material, which is available to authorized users.

## Background

*Bacillus anthracis*, the cause of anthrax in humans and animals, is an endospore-forming bacterium that can persist in the environment for many years. In the U.S., *B. anthracis* infections in animals occur in states that are enzootic for the disease, such as Texas, North and South Dakota, and Minnesota [1–5]. Anthrax outbreaks have occurred annually in livestock and wild game in Texas for the past decade. Outbreaks pertinent to this study include an outbreak in a cattle herd in July 2007 on a ranch north of San Angelo, Texas, and an outbreak in horses on an adjacent ranch in August 2007 [6, 7].

Identification of *B. anthracis* is commonly based on colony morphology (including hemolysis), capsule production, detection of virulence plasmids (pXO1 and pXO2) by real-time PCR, and susceptibility to lysis by gamma phage [8–10]. The gamma phage assay is widely used as a diagnostic test for *B. anthracis* in many laboratories due to ease and low cost of use. Recently, several studies have used gamma phage binding and lytic activity as the basis for newer diagnostic assays, such as using engineered phage for bioluminescent detection [11–16]. The specificity and sensitivity of gamma phage has been previously documented, and although the specificity for *B. anthracis* is very high (97%), a small number of isolates of close relatives, including examples of *Bacillus cereus* and *Bacillus thuringiensis*, are susceptible [9, 17]. In this study, we evaluated the specificity of the gamma phage assay in conjunction with other diagnostic tests to screen 700 spore-forming Texas soil isolates for identification of *B. anthracis*.

## Methods

### Texas soil collection and processing

Core soil samples were collected in September 2007 from two sites in Texas in or near the municipalities of Schertz and San Angelo. Schertz was chosen due to the previous identification of pathogenic *B. cereus* isolates (two clinical, one environmental) harboring *B. anthracis* toxin and/or capsule genes that were associated with fatal pneumonias in metal workers from the area in 2003 [18]. San Angelo was chosen due to the recent anthrax outbreaks in cattle and horses mentioned above [6, 7]. Sterile 50 mL conical tubes were uncapped and twisted down into the soil to a depth of approximately 2.5–3.0 cm. The tubes containing the core samples were then re-capped and wrapped in parafilm. Samples were shipped at ambient temperature and subsequently stored at – 30 °C until processing.

Three samples were selected for testing (one from Schertz and two from San Angelo), and 2–3 bacterial extractions were performed on each sample. For each extraction, approximately 3.5 g of soil were transferred to a sterile 15 mL conical tube. The soil was saturated with phosphate-buffered saline containing 0.3% Tween 20 (PBST) and an additional 1.0 mL of PBST was added, then the sample was vortexed at high speed for 1–3 min. To select for spore-forming bacteria, the samples were heat shocked at 65 °C for 30 min and allowed to settle. The supernatant was transferred to a 2.0 mL screw-cap tube, and briefly centrifuged at 2000–3000 rpm. The following amounts were plated to produce 20–50 colonies/plate: 100 µL of the undiluted supernatant was plated onto PLET (polymyxin, lysozyme, ethylenediaminetetraacetic acid, thallium acetate) agar plates, a selective medium for *B. anthracis* [19], while 100 µL aliquots of a 1:20 dilution were plated onto 10 trypticase soy agar plates containing 5% sheep blood (SBA). Both PLET and SBA plates were incubated overnight at 37 °C. Seven hundred random single colonies were picked and streaked to SBA for isolation. Isolates were then streaked onto sporulation medium (agar containing tryptone, peptone, yeast extract, and manganous chloride) and incubated at 30 °C for 5–7 days. Spores were harvested in 25% glycerol in deionized water and stored as spore suspensions at – 70 °C.

### Isolate screening and identification

Phenotypic characteristics, including hemolysis and colony morphology on SBA (see Additional file 1), and gamma phage susceptibility were recorded for all isolates. Gamma phage testing was performed as previously described [9], with susceptibility defined as any amount of lysis or reduced growth where gamma phage was applied. Cell lysates containing DNA were prepared as previously described [8], and were tested using the Laboratory Response Network’s real-time PCR assay for detection of the *B. anthracis* chromosome, and of virulence plasmids pXO1 and pXO2 genes (*pagA* and *capB*, respectively) [8, 17]. For isolates exhibiting susceptibility to gamma phage lysis, the cell wall (CW) and capsule (CAP) direct fluorescent antibody (DFA) assays were also performed as previously described [10]. Gamma phage-susceptible isolates that were ruled-out for *B. anthracis* using the tests above were identified by 16S rRNA gene sequencing as previously described [20]. For isolates that produced readable sequences, the sequences were compared to published type strain sequences (when applicable) in NCBI’s GenBank database using BLAST, using a sequence similarity of 99–100%. The 16s rRNA sequences of isolates 2008723338, 2008723339, 2008723400, 2008723423, 200872372 and 2008723476 were deposited in GenBank with Accession Numbers KT254134, KT254135, KT254136, KT254137, KT254138 and KT254139, respectively. Isolates identified as belonging to the *B. cereus* group were further typed by multi locus sequence typing (MLST), performed as previously described [21].

## Findings

**Fig. 1:**
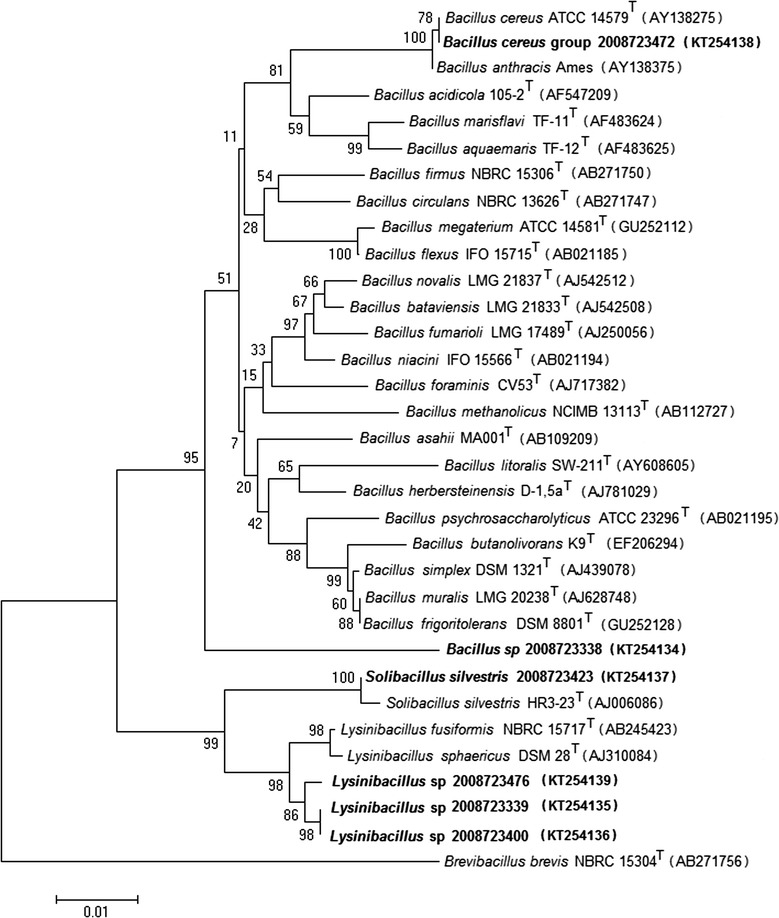
Neighbor-joining dendrogram of 16S rRNA gene sequences, showing the relationship of isolates and representative isolates from each grouping (shown in bold) to a panel of related bacteria. Bootstrap values (based on 1000 replications) are given as percentages at branch nodes. *Brevibacillus brevis* is used as an outgroup for this analysis

Twenty-nine of the 700 isolates were susceptible to gamma phage lysis, with 20 exhibiting hemolysis on SBA and with variable colony morphologies (see Additional files 1 and 2). One isolate was identified as *B. anthracis* (PCR positive for all targets, nonhemolytic, gamma phage-susceptible, and positive by CW- and CAP-DFA assays). The other 28 isolates were negative for all three *B. anthracis* PCR markers, including virulence genes *pagA* and *capB*, and all were negative for the CW-DFA and CAP-DFA assays except for isolate 2008723634, which was weakly positive for the CW-DFA assay. Based on the combined results of these tests, 28 of the 29 gamma phage-susceptible isolates were ruled out as *B. anthracis*. We calculated the specificity of the gamma phage test as 96% by dividing the number of true negatives by the sum of the number of true negatives plus the number of false positives, multiplied by 100 (see Table 1). Twenty-five of these 28 isolates produced readable 16S rRNA gene sequences, and were identified as follows: six *Lysinibacillus* spp., one *Bacillus* sp., one *Solibacillus silvestris*, and 17 isolates could only be identified as “*B. cereus* group members” due to the inability to distinguish members of the *B. cereus* group based on 16S gene sequencing. Representative isolates from each group are shown in Fig. 1. The 16S sequence data was not analyzed for three isolates due to failure to amplify.

**Table 1:** Results of gamma phage susceptibility testing

	Final identification^a^		Totals
	*B. anthracis*	Other spp.	
Gamma phage lysis			
Positive	1^b^	28^c^	29
Negative	0^d^	671^e^	671
Totals	1	699	700

^a^Final identification based on *B. anthracis* real-time PCR, 16S rRNA gene sequencing, and CW- and CAP-DFA

^b^Number of true positives

^c^Number of false positives

^d^Number of false negatives

^e^Number of true negatives

**Fig. 2:**
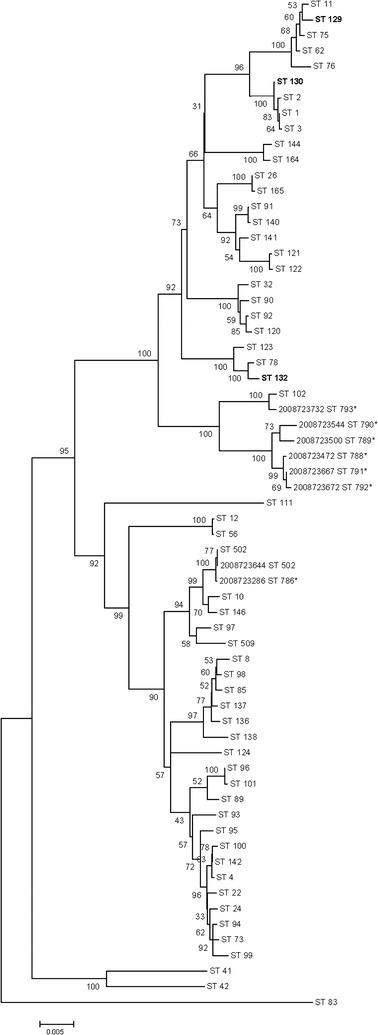
Relationships between *B. cereus* group member isolates of this study and select reference isolates using concatenated sequences from seven housekeeping alleles. Sequence types (STs) with previously identified gamma phage susceptible isolates are shown in bold, and isolates from this study with new STs are marked (asterisk). The tree was constructed using the neighbor joining method and percent bootstrap confidence levels were calculated using 1000 resamplings of the original data

Multi locus sequence typing was performed on all of the 17 *B. cereus* group isolates, however, only eight isolates produced usable gene segments for all seven alleles. All eight isolates were different sequence types (ST), and therefore, not multiple isolations of a single clone. Seven of the eight isolates were new STs, and were compared with other previously identified gamma phage-susceptible *B. cereus* isolates, as well as other *B. cereus*-group STs (Fig. 2) [17, 21]. None of the eight *B. cereus* group isolates identified cluster with previously identified gamma phage-susceptible isolates (ST-129, ST-130, or ST-132), however six of the isolates from this study did cluster closely together (Fig. 2) [21]. The other two *B. cereus* group isolates (2008723644 and 2008723286) were identified as ST-502 or clustered nearest to ST-502, respectively.

In conclusion, the diagnostic tests used in this study showed high specificity (real-time PCR 100% and gamma phage 96%). Interestingly, despite reports of *B. cereus* isolates harboring pXO1 and pXO2 plasmids or some of their genes (*pag*A and *cap*B), these markers were not detected in any of the 700 isolates, except for the single *B. anthracis* isolate [18, 22–24]. Though gamma phage susceptibility is considered specific for *B. anthracis*, rare false positives can occur due to the susceptibility of a few other species. To our knowledge, this is the first report of susceptible isolates outside the *Bacillus* genus. Though the isolates described in this study are of environmental origin rather than clinical, and are often thought of as contaminants in the clinical laboratory, most can be opportunistic pathogens [25]. Therefore, it is important to note their existence, especially when new diagnostic methods are being developed using gamma phage or gamma phage proteins for the detection of *B. anthracis* in clinical or environmental samples [11–16].

## Additional files

**Additional file 1: Table S1.** Colony morphologies of gamma phage-susceptible isolates on SBA after overnight incubation at 37 °C.

**Additional file 2: Table S2.** Isolation, hemolysis, and identification results of gamma phage-susceptible isolates.

## Abbreviations

PCR: polymerase chain reaction
PBST: phosphate-buffered saline containing 0.3% Tween 20
PLET: polymyxin, lysozyme, ethylenediaminetetraacetic acid, thallium acetate
SBA: trypticase soy agar plates containing 5% sheep blood
CW-DFA: cell wall direct fluorescent antibody
CAP-DFA: capsule direct fluorescent antibody
MLST: multi locus sequence typing
ST: sequence type
