# Supplementary material for: Bacillus anthracis gamma phage lysis among soil bacteria: an update on test specificity
Source: BMC Res Notes. 2017 Nov 16;10:598. doi: 10.1186/s13104-017-2919-8 (PMC5691394; doi:10.1186/s13104-017-2919-8)
Supplement: Supplementary file 1 — Additional file 1: Table S1. Colony morphologies of gamma phage-susceptible isolates on SBA after overnight incubation at 37 °C. [file 13104_2017_2919_MOESM1_ESM.docx]

Table S1. Colony morphologies of gamma phage-susceptible isolates on SBA^a^ after overnight incubation at 37°C.

| Identifier | Colony Morphology (Elevation, edge, color and appearance, diameter) |
| --- | --- |
| 2008723158 | Umbonate, irregular/undulate, “fried-egg” like, shiny, 3 mm |
| 2008723286 | Flat, crenate, fine granular, iridescent/opaque, >5 mm |
| 2008723293 | Convex, entire, fine granular, opaque, translucent edges, 2 mm |
| 2008723335 | Umbonate, undulate, smooth, opaque, glossy with translucent edges, 2 mm |
| 2008723336 | Raised, glossy/translucent, spreader |
| 2008723338 | Low convex, entire, crenate, glossy/ translucent with light grey opaque center, 1-3 mm |
| 2008723339 | Low convex, entire, irregular, glossy/translucent, light grey, 1 mm |
| 2008723341 | Flat, crenate, rough/coarse granular, glossy, purple-grey, opaque, 3 mm |
| 2008723373 | Convex, crenate, fine granular almost smooth, glossy, green, opaque, 3mm |
| 2008723388 | Raised, undulate, irregular, smooth, glossy/translucent, light purple, spreader |
| 2008723400 | Entire, irregular, smooth, glossy, light purple, opaque, spreader |
| 2008723407 | Low convex, entire, smooth, glossy, light grey, opaque, 2 mm |
| 2008723423 | Raised, entire, smooth, glossy/translucent, light grey, 1-3 mm |
| 2008723425 | Low convex, slightly crenate, fine granular, green, opaque, 3 mm |
| 2008723472 | Umbonate, crenate, granular with smooth center, greenish/opaque, 4 mm |
| 2008723476 | Swarmer, translucent purple-grey, lightly textured |
| 2008723486 | Low umbonate, crenate, granular with smooth center, green, opaque, >5 mm |
| 2008723499 | Umbonate, entire, almost smooth, tan-green, opaque, 4 mm |
| 2008723500 | Slightly crenate, low convex, fine granular, opaque, "bulls eye", 3-4 mm |
| 2008723529 | Low convex, slightly crenate, smooth with button center, green, opaque, 4 mm |
| 2008723532 | Raised, irregular edges, fine granular, green, opaque, 6 mm |
| 2008723544 | Convex, entire, fine granular (almost smooth), opaque, green, 3 mm |
| 2008723548 | Umbonate, undulate, translucent edges, opaque white center, smooth, glossy, 2 mm |
| 2008723634 | Flat, coarsely granular, entire, dull green, >5 mm |
| 2008723644 | Flat, coarsely granular, entire, dull green, >5 mm |
| 2008723667 | Umbonate, crenate, fine granular edges, shiny greenish smooth center, 4 mm |
| 2008723672 | Umbonate, crenate, fine granular edges, shiny greenish smooth center, 4 mm |
| 2008723732 | Slightly umbonate, irregular, fine granular, grey-green with cloudy edges, >5 mm |
| 2008723733 | Slightly umbonate, irregular, fine granular, grey-green with cloudy edges, somewhat shiny, 3 mm |

a SBA, trypticase soy agar plates containing 5% sheep blood.
