# Supplementary material for: Bacillus anthracis gamma phage lysis among soil bacteria: an update on test specificity
Source: BMC Res Notes. 2017 Nov 16;10:598. doi: 10.1186/s13104-017-2919-8 (PMC5691394; doi:10.1186/s13104-017-2919-8)
Supplement: Supplementary file 2 — Additional file 2: Table S2. Isolation, hemolysis, and identification results of gamma phage-susceptible isolates. [file 13104_2017_2919_MOESM2_ESM.docx]

Table S2. Isolation, hemolysis, and identification results of gamma phage-susceptible isolates.

| Soil Sample | Isolation Medium^a^ | Identifier | Hemolysis on SBA^a^ | Identification^b, d^ |
| --- | --- | --- | --- | --- |
| Schertz #1 (SC-1) | PLET | 2008723158 | Yes | Not *B. anthracis*^c^ |
|  | SBA | 2008723286 | No | *B. cereus* group member |
|  |  | 2008723634 | Yes | *B. cereus* group member |
|  |  | 2008723644 | Yes | *B. cereus* group member |
| San Angelo #1 (SR-9) | PLET | 2008723293 | Yes | Not *B. anthracis*^c^ |
|  |  | 2008723667 | Yes | *B. cereus* group member |
|  | SBA | 2008723335 | Yes | *B. cereus* group member |
|  |  | 2008723336 | No | *Lysinibacillus* sp. (Grouping 1) |
|  |  | 2008723338 | No | *Bacillus* sp. |
|  |  | 2008723339 | No | *Lysinibacillus* sp. (Grouping 2) |
|  |  | 2008723341 | No | *B. anthracis* |
|  |  | 2008723373 | Yes | *B. cereus* group member |
|  |  | 2008723388 | No | *Lysinibacillus* sp. (Grouping 1) |
|  |  | 2008723400 | No | *Lysinibacillus* sp. (Grouping 1) |
|  |  | 2008723407 | No | *Lysinibacillus* sp. (Grouping 2) |
|  |  | 2008723423 | No | *Solibacillus silvestris* |
|  |  | 2008723425 | Yes | Not *B. anthracis*^c^ |
|  |  | 2008723472 | Yes | *B. cereus* group member |
|  |  | 2008723476 | Yes | *Lysinibacillus* sp. (Grouping 3) |
|  |  | 2008723486 | Yes | *B. cereus* group member |
|  |  | 2008723672 | Yes | *B. cereus* group member |
| San Angelo #2 (SR-17) | PLET | 2008723499 | Yes | *B. cereus* group member |
|  |  | 2008723500 | Yes | *B. cereus* group member |
|  |  | 2008723529 | Yes | *B. cereus* group member |
|  |  | 2008723732 | Yes | *B. cereus* group member |
|  |  | 2008723733 | Yes | *B. cereus* group member |
|  | SBA | 2008723532 | Yes | *B. cereus* group member |
|  |  | 2008723544 | Yes | *B. cereus* group member |
|  |  | 2008723548 | Yes | *B. cereus* group member |

a PLET, polymyxin, lysozyme, ethylenediaminetetraacetic acid, thallium acetate agar plates; SBA, trypticase soy agar plates containing 5% sheep blood.

b Based on 16S rRNA gene sequencing.

c “Not *B. anthracis”* are the isolates that did not produce 16S rRNA gene sequences, but were ruled out for *B. anthracis* using diagnostic tests.

d Grouping 1, 2, and 3: Three different *Lysinibacillus* spp. identified. Isolates within each grouping have the same 16S sequences and are therefore identified as the same *Lysinibacillus* sp.
